# Do Complexity Measures of Frontal EEG Distinguish Loss of Consciousness in Geriatric Patients Under Anesthesia?

**DOI:** 10.3389/fnins.2018.00645

**Published:** 2018-09-20

**Authors:** Sarah L. Eagleman, Don A. Vaughn, David R. Drover, Caitlin M. Drover, Mark S. Cohen, Nicholas T. Ouellette, M. Bruce MacIver

**Affiliations:** ^1^Department of Anesthesiology, Perioperative and Pain Medicine, Stanford University, Palo Alto, CA, United States; ^2^UCLA Semel Institute for Neuroscience and Human Behavior, Los Angeles, CA, United States; ^3^Department of Psychology, University of Santa Clara, Santa Clara, CA, United States; ^4^University of Washington, Seattle, WA, United States; ^5^UCLA Departments of Psychiatry, Neurology, Radiology, Psychology, Biomedical Physics and Bioengineering, California Nanosystems Institute, Los Angeles, CA, United States; ^6^Department of Civil and Environmental Engineering, Stanford University, Stanford, CA, United States

**Keywords:** anesthesia, geriatric, electrophysiology (EEG), propofol, fentanyl, nonlinear dynamics, multiscale entropy, 1/*f*

## Abstract

While geriatric patients have a high likelihood of requiring anesthesia, they carry an increased risk for adverse cognitive outcomes from its use. Previous work suggests this could be mitigated by better intraoperative monitoring using indexes defined by several processed electroencephalogram (EEG) measures. Unfortunately, inconsistencies between patients and anesthetic agents in current analysis techniques have limited the adoption of EEG as standard of care. In attempts to identify new analyses that discriminate clinically-relevant anesthesia timepoints, we tested 1/*f* frequency scaling as well as measures of complexity from nonlinear dynamics. Specifically, we tested whether analyses that characterize time-delayed embeddings, correlation dimension (CD), phase-space geometric analysis, and multiscale entropy (MSE) capture loss-of-consciousness changes in EEG activity. We performed these analyses on EEG activity collected from a traditionally hard-to-monitor patient population: geriatric patients on beta-adrenergic blockade who were anesthetized using a combination of fentanyl and propofol. We compared these analyses to traditional frequency-derived measures to test how well they discriminated EEG states before and after loss of response to verbal stimuli. We found spectral changes similar to those reported previously during loss of response. We also found significant changes in 1/*f* frequency scaling. Additionally, we found that our phase-space geometric characterization of time-delayed embeddings showed significant differences before and after loss of response, as did measures of MSE. Our results suggest that our new spectral and complexity measures are capable of capturing subtle differences in EEG activity with anesthesia administration—differences which future work may reveal to improve geriatric patient monitoring.

## Introduction

About 50% of geriatric patients aged 65 and older will require anesthesia for a surgical procedure at some time in their remaining years (Kim et al., 2015). It has been suggested that geriatric patients have an increased risk of dementia, delirium, and neurocognitive dysfunction after exposure to anesthetic agents (Avidan and Evers, 2011; Chen et al., 2013; Strøm et al., 2014; Purdon et al., 2015a; Yang and Fuh, 2015). Given this potential risk, several investigators have suggested that geriatric patients may benefit from maintenance at lighter anesthetic levels (Lindholm et al., 2009; Kalkman et al., 2011; Strøm et al., 2014; Petsiti et al., 2015), which may reduce the risks of developing dementia (Chen et al., 2014). However, a downside of light anesthesia is the possibility that a patient may not be unconscious during the procedure. It is estimated that 1–2 out of every 1000 patients undergoing general anesthesia for surgery are insufficiently anesthetized, and yet are immobilized and unable to respond (Bischoff and Rundshagen, 2011). There would be tremendous advantage in the ability to titrate an anesthetic dose accurately to balance an improved medical outcome against the risk of intraoperative awareness.

Electroencephalogram (EEG) signals, especially from the frontal cortex, exhibit stereotypical responses to some anesthetics. For example, loss of consciousness (LOC) correlates with a transition from low amplitude, high frequency EEG waveforms to high amplitude, low frequency patterns, resembling the transition to sleep. In anesthesia, low frequency delta (∼1–4 Hz) rhythms are replaced gradually by burst suppression patterns as patients transition to deeper surgical planes of anesthesia (Pilge et al., 2014). Since the 1990s, several monitoring devices have been developed to capitalize on these EEG frequency domain transitions (Fahy and Chau, 2018). Unfortunately, the resultant measures are inconsistent as EEG changes depend on the anesthesia and the patient (Akeju et al., 2015; Purdon et al., 2015a,b).

In particular, geriatric patients pose a unique challenge for electrophysiological monitoring because brain activity (as measured with EEG) attenuates with advancing age (Akeju et al., 2015; Purdon et al., 2015b; Lee et al., 2017). This makes it difficult to quantify the frequency changes between awake and anesthetized states accurately in individual patients. None of the EEG intraoperative monitoring methods account for the differences that exist in the geriatric population. In addition, geriatric surgical patients are often medicated with beta-adrenergic blockers (beta-blockers). Beta-blockers may hide the cardiovascular signs of inadequate anesthesia (tachycardia and hypertension) (Ghosh et al., 2008), and geriatric patients treated with beta-blockers may require less anesthesia (Zaugg et al., 2003; Ghosh et al., 2008). Importantly, beta-blockers themselves can cause changes in the EEG activity, and thus are likely to cause problems with anesthetic depth monitoring (Johansen, 2001; Zaugg et al., 2003; Ghosh et al., 2008). The result is that EEG monitoring has not been adopted as standard of care. The development of efficacious, anesthetic- and patient-invariant EEG processing techniques could mitigate this and provide a better way to monitor patients and improve outcomes. Thus, in the current study, we analyzed EEG data collected from geriatric patients who received beta-blockers for at least 24 h prior to surgery, and who were subsequently anesthetized with fentanyl and propofol.

Fentanyl and propofol are used in combination routinely to obtain balanced anesthesia for induction during surgical procedures. Fentanyl is a potent opioid that decreases the intensity of response to intubation and provides general pain relief during surgical procedures. Administration of fentanyl correlates with a shift in the EEG pattern from high frequency, low amplitude to low frequency, high amplitude (Scott et al., 1985). Propofol has been shown to increase frontal EEG activity in the alpha (8–14 Hz) and slow (0.1–1 Hz) bands after LOC (Gugino et al., 2001; Feshchenko et al., 2004; Purdon et al., 2013; Akeju et al., 2014). This change in activity occurs in both young and elderly patients (Purdon et al., 2015a); however, the reduction in amplitude with age causes more subtle differences in spectral characteristics with anesthesia onset, which may not be detected by modern EEG monitoring devices (Purdon et al., 2015b).

A potentially more sensitive spectral measure of anesthetic depth might be 1/*f*. Electrophysiological signals demonstrate 1/*f*-like frequency scaling (He, 2011): the power declines relative to increases in frequency composition. Importantly, this measure has demonstrated sensitivity to the electrophysiological brain state changes associated with sleep (Bédard et al., 2006). Further, 1/*f* scaling in EEG changes with age (Voytek et al., 2015). To our knowledge, 1/*f* frequency scaling has not been tested on EEG protocols that include anesthesia.

In addition to spectral measurements, previous studies have demonstrated that complexity measures from nonlinear dynamics correlate with anesthetic depth (Watt and Hameroff, 1988; Widman et al., 2000; van den Broek et al., 2006; Walling and Hicks, 2006; MacIver and Bland, 2014; Eagleman et al., 2018). Structural changes in time-delayed embeddings (attractors) of EEG signals have been reported in both rodents (MacIver and Bland, 2014) and humans (Watt and Hameroff, 1988; Widman et al., 2000; Walling and Hicks, 2006; Eagleman et al., 2018). The awake attractors appear in 3D as spheroids, and then flatten to ellipsoids with LOC (Watt and Hameroff, 1988; Walling and Hicks, 2006; MacIver and Bland, 2014; Eagleman et al., 2018). Previous work has quantified these attractor changes using the correlation dimension (CD) (Grassberger and Procaccia, 1983; Widman et al., 2000; Walling and Hicks, 2006) and a phase-space geometric fit called the ellipse radius ratio (ERR), which fits the attractor with an ellipsoid solid of revolution, and then reports the ratio of the lengths of the minimum and maximum symmetry axes (Eagleman et al., 2018). Another complexity measure, multiscale entropy (MSE), has also been reported to correlate well with existing anesthetic depth measures (Li et al., 2010; Liu et al., 2015). In the current study, we tested whether these complexity measures could distinguish between subtle changes in EEG activity occurring before and after LOC.

We performed a retrospective analysis of data collected from geriatric patients (aged 65 and older) who were on beta-adrenergic blockers at least 24 h prior to surgery. Patients were anesthetized with fentanyl and propofol. We identified and analyzed 20 s clips of frontal EEG from before and after loss of response to verbal commands (LOR, considered here as loss of consciousness). We compared frequency-derived measures, as well as complexity measures, of the EEG signal in the before-and-after clips to identify measures that discriminated between the two states.

## Materials and Methods

### Study Protocol

All procedures took place under an approved protocol from the Stanford School of Medicine Administrative Panel on Human Subjects in Medical Research (ClinicalTrials.gov, NCT00938782). The database consisted of 67 surgical patients all older than 65 years of age. Patients underwent anesthesia for non-cardiac procedures classified as status 1–3 by the American Society of Anesthesiologists. All patients in the original study were receiving beta-adrenergic treatment for a minimum of 24 h preoperatively and received their medication prior to surgery. We reviewed the database retrospectively and analyzed data under a separate Stanford-approved IRB. Out of the 67 patients, 28 patients were used for analysis (details of selection below). These 28 patients had an average age of 76 (± 6) years and included 18 males and 10 females. They had an average body mass index (BMI) of 27.3 (± 4.7). The healthy BMI range for patients aged 65 and older has been suggested as 23 to 30 (Porter Starr and Bales, 2015). Our analysis included 8 patients with a BMI greater than 30 and 5 patients with a BMI less than 23.

The surgical procedures and anesthetic administration have been described in detail previously (Drover et al., 2011). Briefly, patients were induced with fentanyl (1–3 mcg/kg), propofol (1–2 mg/kg), and muscle relaxant (if required) using either rocuronium (0–1 mg/kg) or vecuronium (0.1 mg/kg). After intubation, sevoflurane in oxygen with 50–60% nitrous oxide was initiated and used to maintain anesthesia. Medical staff recorded significant clinical events including induction, anesthetic administration and dosage, LOR to verbal commands, and time.

The average time of fentanyl use prior to LOR was 2.1 ± 1.6 min. With propofol, the average before LOR was 1.0 ± 0.6 min. A total of 6 patients received muscle relaxant before LOR. One patient received vecuronium 0.7 min before LOR, and 5 patients received rocuronium an average of 5.2 min before LOR. A total of 14 patients received muscle relaxant within a 4 min window following LOR. Three patients received vecuronium and 11 received rocuronium, both an average of 0.8 min following LOR. In addition, 18 patients were started on sevoflurane in oxygen with 50–60% nitrous oxide an average of 1.7 min following LOR.

### EEG Recording and Preprocessing

**FIGURE 1:**
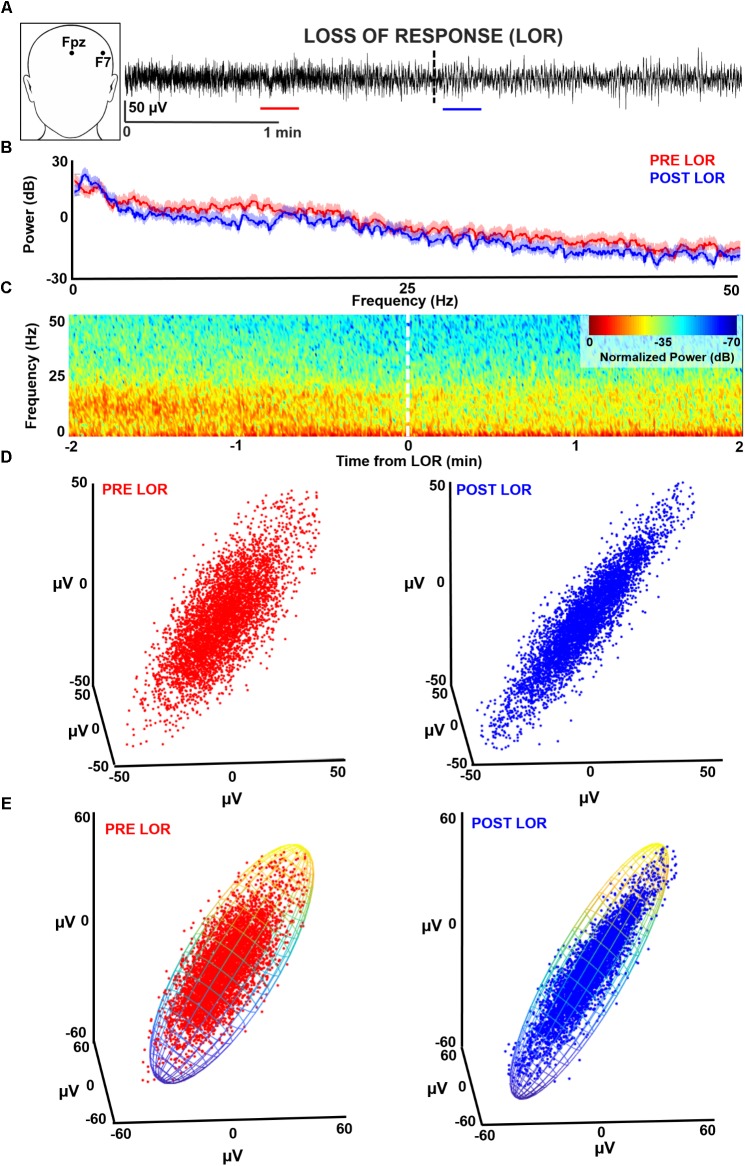
Characterizing loss of response (LOR) using electroencephalogram (EEG) analysis. **(A)** EEGs were recorded from F7 during induction with propofol and fentanyl anesthesia in geriatric patients receiving beta-adrenergic blockade. We selected continuous artifact-free 20 s clips from before (red) and after (blue) patients lost response to verbal commands. **(B)** We analyzed these clips using multitaper spectral analysis. In this patient (PT09) an increase in lower frequencies and a decrease in higher frequencies from before to after LOR can be observed. **(C)** To view the dynamics of the spectral changes through time we plotted a normalized spectrogram starting 2 min before LOR to 2 min after LOR. We can see the increase in lower frequencies and decrease in higher frequencies coordinates well with the LOR timestamp. **(D)** We tested several complexity measures of time-delayed embeddings (attractors). An awake attractor (red) looks less ellipsoidal than the anesthetized attractor (blue). **(E)** One method we used to quantify change in attractors before and after LOR is by fitting the attractor with an ellipsoid solid of revolution. We then calculate the ratio of the minimum and maximum radii.

Electroencephalogram recordings were acquired using a SedLine Legacy EEG monitor (Masimo, Irvine, CA, United States). The manufacturer’s standard adhesive electrode was attached to the patient prior to starting the anesthetic, as per the manufacturer’s instructions. EEGs were recorded at approximately F7 grounded to Fpz and referenced to ∼1 cm above Fpz (**Figure 1A**). Data was digitized at 250 Hz. Records of the surgical events including time stamps of start of induction, LOR to verbal stimuli, and administration and dosages were de-identified and then used for analysis. The EEG recordings were de-trended, and notch filtered using a second-order Butterworth Infinite Impulse Response (IIR) filter to remove 60 and 120 Hz noise prior to analysis. Additionally, a second-order Butterworth IIR filter was used to remove a 78.125 Hz impedance measurement pulse generated by the EEG monitor system.

We identified each subject’s 20 s pre-LOR and post-LOR EEG clip via a two-step algorithm which was a mix of exclusion and inclusion criteria (**Figure 1A**). First, three of the authors (SLE, DRD, and MBM) visually inspected the EEG traces, spectrums, processed spectrograms to identify EEG periods containing burst suppression or artifacts (exclusion criteria). Second, given the fundamentally imprecise metric of LOR, from these remaining clips we used the clips most temporally-distant from the LOR timepoint (within a 2 min window) to obtain the clips most representative of pre-LOR and post-LOR (inclusion criteria). From the original 67 patients, 28 were selected who had LOR timestamps and artifact and noise free EEG clips for at least 20 continuous seconds before and after LOR.

### Spectral Analyses

To visualize the spectral changes that occurred in our clips before and after LOR, we performed multitaper spectral analysis on the 20 s clips during pre and post LOR period using the MATLAB Chronux toolbox (**Figure 1B**; Mitra and Bokil, 2008).^1^ Specifically, we used a time-bandwidth product of 5 with 9 tapers, limited the frequency ranges calculated to 0 to 50 Hz, and computed the theoretical error range at the 95% confidence interval. Power values were expressed in decibels.

To give an example of the temporal profile of the spectral changes that occurred during the windows surrounding the LOR transitions, we computed a normalized spectrogram (**Figure 1C**). We calculated the Fourier transform using Hann windows with half window overlaps. We then cutoff the frequencies above 50 Hz, converted the magnitude to decibels (dB), and scaled the spectrogram output by its maximum magnitude.

We calculated the spectral edge frequency (i.e., the frequency bounding 95% of the power from above) and total power using multitaper spectral analysis (Mitra and Bokil, 2008) without limiting the frequency range to below 50 Hz. We calculated the percentage of total power for individual frequency bands per condition. The percentage of total power was used because of prior reports of significant changes in total power with exposure to anesthetics. The ranges we used for the frequency bands were as follows: delta: 0.1 to 4 Hz; theta: 4 to 8 Hz; alpha: 8 to 14 Hz; beta: 14 to 30 Hz; and gamma: above 30 Hz (Gugino et al., 2001; Purdon et al., 2013). To test whether we observed similar changes in before and after clips compared to other studies, we calculated the slow frequency component (0.1–1 Hz) separately.

To determine whether 1/*f* characteristics change before and after LOR, we fit each patient’s spectral power to *c/f^α^*, where *α* and *c* were free parameters representing the quickness of frequency decay and an arbitrary constant, respectively. We determined *α* and *c* for each patient by minimizing the L_2_ norm of the residuals between predicted and actual values.

### Multiscale Entropy

As complex signals often have meaningful relationships at multiple timescales, we used MSE to characterize the relationship and complexity of the EEG time series. MSE utilizes an algorithm that calculates a traditional entropy metric at several timescales (Costa et al., 2002). In this case, we used sample entropy as our multiscale metric (Pincus, 1991) as it has been shown to extract meaningful complexity differences in a variety of physiological signals (Costa et al., 2003; Bian et al., 2009; Liu et al., 2013). We calculated MSE using custom MATLAB (The MathWorks, Inc.) scripts.

### Characterization of Dynamical Attractors

#### Correlation Dimension

We constructed three-dimensional time-delayed embeddings (attractors) of the EEG signal before and after LOR using an 8 ms delay (**Figure 1D**) as previously described (Watt and Hameroff, 1988; Walling and Hicks, 2006; MacIver and Bland, 2014; Eagleman et al., 2018). We chose this delay as we observed shape changes in attractors when plotted at this timescale. We tested whether the CD captured this shape change with LOR. CD is used to determine the non-integer (fractal) dimensionality of irregular objects (e.g., a point cloud in our case) as described previously (Grassberger and Procaccia, 1983; Widman et al., 2000; Walling and Hicks, 2006). We also tested whether significant differences in CD could be observed when we increased the dimensionality of the embeddings from 3D to 5D.

We tested whether the embedding delay of the attractor impacted the estimate of the CD value. To estimate the optimal delay for creating the attractors we calculated the first zero-crossing of the autocorrelations of the pre-LOR and post-LOR signals. This is the first time lag that the EEG signal differs maximally with itself. We used this value to set our maximum range of embedding delays and tested multiple delays between the shortest time window (shifting the EEG by 1 point) to the largest (set by the autocorrelation zero crossing). We tested delays of 4, 8, 12, 52, 100, 500, 1000, 1500, 2000, and 2500 ms. We tested these delays for both 3D and 5D time-delayed embeddings. We tested whether a trend existed between embedding delay and CD using the Spearman correlation.

#### Ellipse Radius Ratio

We constructed three-dimensional time-delayed embeddings, as described above (**Figure 1D**). We quantified this shape change by fitting the three-dimensional attractor to an ellipsoidal solid of revolution (**Figure 1E**; Khachiyan, 1980; Eagleman et al., 2018). The lengths of the symmetry axes of the ellipsoid were calculated and the ratio of the minimum and maximum axes (which we term the ellipsoid radius ratio, ERR) was used to quantify the shape change. A radius ratio of 1 implies a sphere, while smaller ratios imply more strongly ellipsoidal shapes.

Similar to the CD, we tested also whether the ERR was changed by the embedding delay time. We created time delayed embeddings using the same delays as were used to calculate the CD. We tested whether a trend existed between embedding delay and ERR using the Spearman correlation statistic.

### Correlations Between EEG Measures and Effect Size of EEG Measures

To test whether our EEG measures correlated with patient age or body-mass index (BMI), we calculated the Spearman correlation statistic between the change in ERR (LORpost–LORpre) at the shortest delay (4 ms) with corresponding patient ages and BMIs. We did the same for the MSE results.

To test whether our EEG measures correlated with each other or with spectral changes, we calculated the Spearman correlation statistic between the changes in these measures before and after LOR. Specifically, we tested the correlation between changes in ERR and MSE as well as between ERR or MSE and percentage of power change (LORpost–LORpre) in the individual frequency bands that we measured (delta, theta, alpha, beta, and gamma). For the change in ERR, we chose the values calculated at the shortest delay (4 ms) as these showed the most significant changes before and after LOR.

We calculated a paired-data Cohen’s D on our EEG measures before and after LOR for the novel spectral (1/*f*) and complexity (MSE and ERR phase space analysis) measures that showed significant differences before and after LOR. We also calculated Cohen’s D for the percentage of power in each of the frequency bands (delta, theta, alpha, beta, and gamma) for comparison.

### Statistics

We corrected significance values for multiple univariate statistical comparisons within a particular analysis type, by using the Holm-Bonferroni method—a sequentially-rejective procedure (Holm, 1979). Specifically, we corrected *p* values for pre vs post metrics within each of the following analyses: (1) the power percentage across all 5 frequency bands (delta, theta, alpha, beta, gamma), (2) the CD across all 10 embedding delays, (3) the ERR across all 10 embedding delays, and (4) the correlation of MSE, ERR, and frequency band power. We report our results as medians (25, 75 percentiles), and significance values (*p*) are calculated from Wilcoxon Signed Rank Tests.

## Results

### Spectral Analyses

**FIGURE 2:**
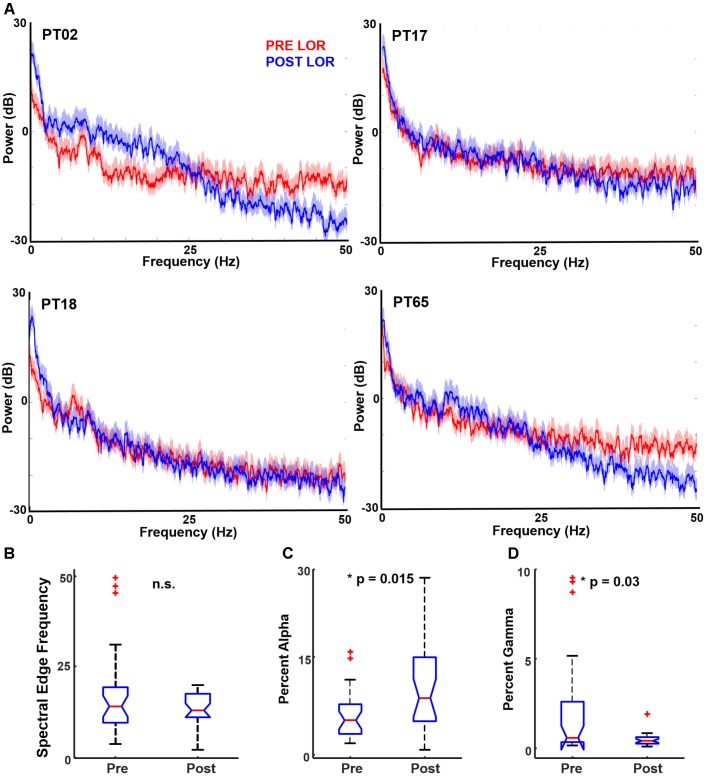
Spectral characteristics before and after loss of response (LOR). **(A)** Example of multitaper spectrum from four patients pre- (red) and post- (blue) LOR. Note the increases in alpha and decrease in gamma activity following LOR. The shaded region represents the 95% confidence interval. We computed several standard spectral measures pre- and post-LOR. **(B)** Spectral edge frequency has been shown to correlate with anesthetic depth; however, we did not observe a significant difference. **(C)** We observed a significant increase in alpha activity after LOR (*p* = 0.015 corrected). **(D)** We observed a significant decrease in gamma activity after LOR (*p* = 0.03 corrected).

We performed multitaper spectral analysis to quantify the changes that occur before and after LOR (**Figure 2A**). To compare our measures to previous reports, we computed a common spectral measure that correlates with anesthetic depth: the spectral edge frequency. We also tested whether total power differed from before and after LOR. We did not observe a significant change in spectral edge frequency before and after LOR (**Figure 2B**, pre-LOR 14.1 Hz [9.6, 19.4], post-LOR 13.0 Hz [11.1, 17.6], *p* = 0.64). We did not observe a significant difference in total power before and after LOR (pre-LOR 35.2 dB [33.0, 36.2], post-LOR 34.6 dB [32.8, 36.3], *p* = 0.21).

To determine the changes in frequency bands, we calculated the changes in delta (0.1–4 Hz), theta (4–8 Hz), alpha (8–14 Hz), beta (14–30 Hz), and gamma (above 30 Hz) ranges. Significant differences were found for alpha (**Figure 2C**) and gamma (**Figure 2D**), and all spectral results are summarized in **Table 1**. Similar to previous reports, we observed a significant increase in alpha from before to after LOR. In addition, we observed a significant decrease in gamma power from before to after LOR. In a separate analysis, we separated out the slow (0.1–1 Hz) frequency component from the EEG signal to see if the percentage of slow activity changed before to after LOR. We did not observe a significant difference in this frequency band (pre-LOR 48.6% [37.6, 62.9], post-LOR 54.3% [34.0, 64.4], *p* = 0.57).

**Table 1:** Summary of spectral changes from before and after loss of response (LOR).

Frequency	Percentage	Percentage	Significance
band	of power	of power	value,
	PRE LOR	POST LOR	corrected
Delta (0.1–4 Hz)	76.3% [66.5, 83.7]	74.1% [60.1, 82.8]	0.22
Theta (4–8 Hz)	4.8% [3.2, 7.1]	6.4% [4.3, 9.4]	0.34
Alpha (8–14 Hz)	5.3% [3.2, 7.7]	8.7% [5.1, 15.0]	0.015^∗^
Beta (14–30 Hz)	3.8% [2.2, 5.3]	3.9% [2.8, 8.9]	0.26
Gamma (>30 Hz)	0.6% [0.4, 2.6]	0.4% [0.3, 0.6]	0.03^∗^

*Percentage of power in individual frequency bands are reported as medians [25, 75 percentiles]. Significance values reported are from Wilcoxon signed rank tests, corrected for multiple comparisons. The ^∗^ indicates the percentage of power change was significant at *p* < 0.05 corrected*.

**FIGURE 3:**
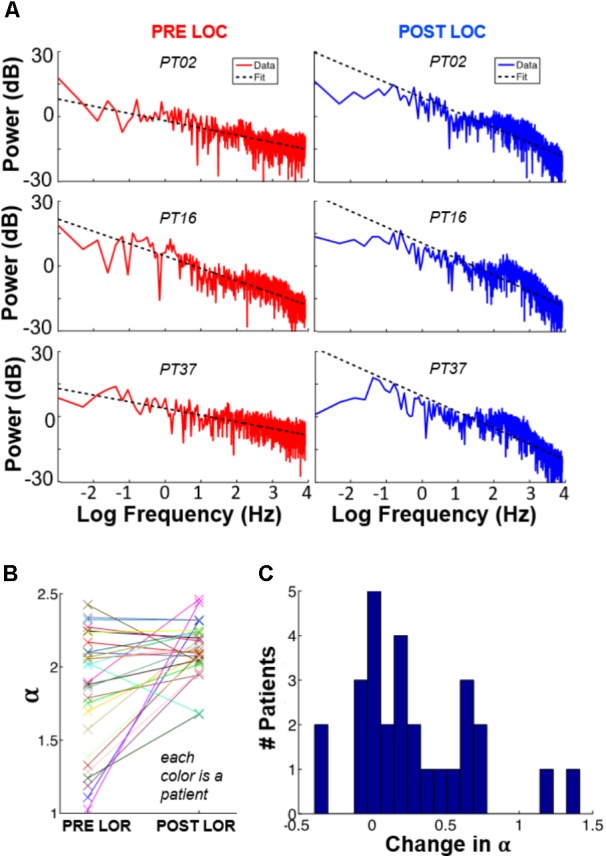
1/*f* spectral changes occur with loss of response. We fit each patient’s spectral power to c/*f^α^*, where *α* and *c* were free parameters representing the quickness of frequency decay and an arbitrary constant, respectively. Panel **(A)** shows these plots for three patients, before and after LOC. **(B)** Values of *α* for each subject, pre-LOR and post-LOR. **(C)** Overall, we observed that 71% of the patients show an increase in *α* after LOR.

To determine whether 1/*f* characteristics change before and after LOR, we fit each patient’s spectral power to *c/f^α^* (**Figure 3A**). Overall, 71% of the patients showed an increase in the value of *α* from pre-LOR to post-LOR (**Figure 3B**). This difference in *α* before and after LOR differed significantly from the null hypothesis of no change (median change = 0.17, *p* < 10^-3^, **Figure 3C**).

### Characterization of Dynamical Attractors

**FIGURE 4:**
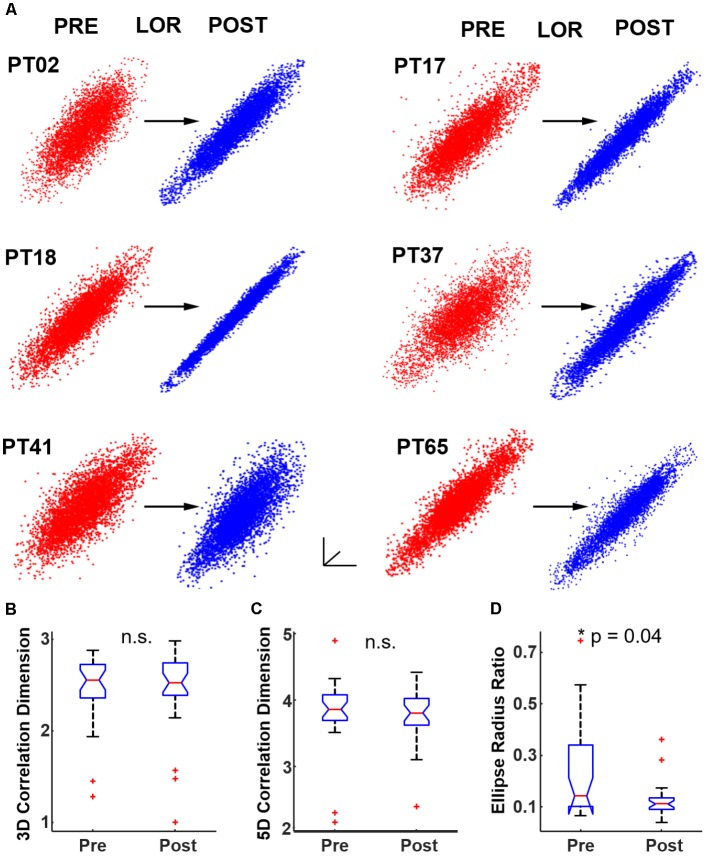
Quantifying dynamical attractors before and after loss of response (LOR). **(A)** We created time-delayed embeddings (attractors) from 20 s continuous clips. A shape change from thicker, less-ellipsoidal attractors before LOR (red) to flatter, more ellipsoidal attractors after LOR (blue) was observed and is shown here for 6 patients. We quantified the difference in attractors using both correlation dimension and our ellipse radius ratio (ERR). **(B)** We did not observe a significant difference in correlation dimension in 3 dimensions, nor did we observe a significant difference in correlation dimension in 5 dimensions **(C)**. **(D)** We did observe a significant difference in our ERR measure from before to after LOR (*p* = 0.04 corrected). The ^∗^ indicates significant difference between pre-LOR and post-LOR measures at *p* < 0.05 corrected.

We began by testing whether significant differences could be observed in CD between 3D attractors plotted at an 8 ms embedding delay. We observed a similar flattening of the attractor and more ellipsoidal shapes after LOR (**Figure 4A**). We quantified the pre- to post-LOR changes in 3D attractors using CD. We did not observe a significant difference using this measure (**Figure 4B**). We also calculated the CD for 5 dimensional attractors. Here again we did not observe a significant difference pre- and post-LOR (**Figure 4C**).

We then tested our ERR with the 8 ms attractors before and after LOR. We found a significant difference between pre- and post-LOR (**Figure 4D**, *p* = 0.04 corrected).

**FIGURE 5:**
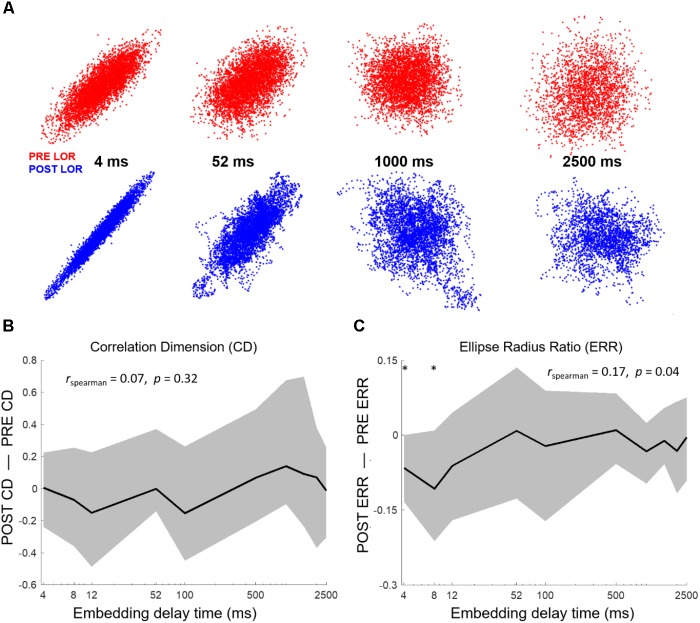
Attractor shape changes are embedding delay dependent. **(A)** Attractors from PT02 shown at 4, 52, 1000, and 2500 ms. The shape change in the attractor is not observed at higher embedding delays. We tested whether our attractor characterization analyses correlated with embedding delay. We computed correlation dimension and ellipse radius ratio (ERR) at 10 different delays from 4 to 2500 ms. **(B)** No significant changes in correlation dimension were observed from before to after LOR for any embedding delay nor was correlation dimension correlated with the embedding delay. Here we show results from the 3D correlation dimension calculation. **(C)** We observed a significant correlation between the ERR and embedding delay (r_spearman_ = 0.17, *p* = 0.04) and a significant difference in the ERR between pre- and post-LOR for 4 ms and 8 ms delays (*p*_4_ = 0.017 corrected, *p*_8_ = 0.04 corrected). The ^∗^ indicates a significant difference between the measured values before and after loss of response at that embedding delay at *p* < 0.05 corrected.

To test the impact of the embedding delay on the attractor calculations we chose multiple embeddings delays between the smallest possible delay (4 ms, shifting the EEG by 1 point) and the largest (set by the first zero-crossing of the autocorrelation). We calculated the first zero-crossing for both the pre- and post-LOR period to see if there was a difference. We did not find a significant difference between the pre- and post-LOR values (pre-LOR 1800 ms [1392, 2856], post-LOR 2176 ms [1664, 2480], *p* = 0.35 corrected). Given these results, we decided to calculate CD using the following embedding delays: 4, 8, 12, 52, 100, 500, 1000, 1500, 2000, and 2500 ms. The impact of embedding delay on attractor shape is shown in **Figure 5A**. We did not observe a significant difference at any delay for 3D or 5D CD calculations, nor did we observe a significant correlation between CD and embedding delay (**Figure 5B**).

We performed the same test of different embedding delays with our ERR analysis. The ERR showed a significant positive relationship with the difference in post-LOR–pre-LOR conditions (**Figure 5C**, *p* = 0.04, Spearman correlation). This relationship likely was driven by a reduction in the ERR between the pre- and post-LOR states at short embedding delays: only embedding delays of only 4 and 8 ms showed a significant difference (*p_4_* = 0.017 corrected, *p_8_* = 0.04 corrected).

### Multiscale Entropy

**FIGURE 6:**
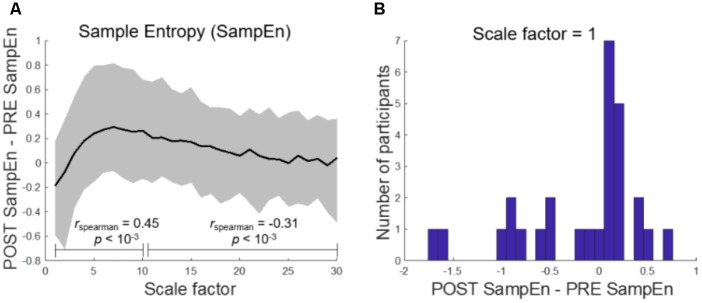
Multiscale entropy reveals scale-dependent complexity change after loss of response. **(A)** Sample Entropy (SampEn) shows a decrease in complexity at short scale factors which monotonically increases until medium scale (r_spearman_ = 0.45, *p* < 10^-3^) and then decreases toward 0 (r_spearman_ = -0.31, *p* < 10^-3^). Here, the solid black line represents the mean. **(B)** At the shortest scale, the difference in SampEn between post-LOR and pre-LOR conditions is not significantly different from chance as measured by the median, but is when measured by mean (*p* = 0.04, percentile permutation test).

A MSE analysis between the pre-LOR and post-LOR conditions revealed a scale-dependent change in sample entropy (**Figure 6A**). Complexity decreased at short scale factors, showing a statistically significant trend toward increasing at medium scale factors (*r_spearman_* = 0.45, *p* < 10^-3^ percentile permutation test). The difference in sample entropy between post and pre conditions then showed a decreasing relationship with a further increase in scale factor (*r_spearman_* = -0.31, *p* < 10^-3^ percentile permutation test). The initial decrease in complexity at a scale factor of 1, which was not significantly different by median (*p =* 0.48), likely resulted from sharp decreases in MSE among several of the participants (**Figure 6B**).

### Correlations Between EEG Measures and Effect Size of EEG Measures

Spearman correlation revealed no significant relationship in our ERR or MSE changes before and after LOR and with patient age or BMI (**Table 2**). Spearman correlation between our ERR (calculated at the shortest delay, 4 ms) and MSE changes before and after LOR revealed that they are correlated with each other (**Table 3**, *r* = 0.54, *p* = 0.0034 corrected). Additionally, a significant correlation between ERR change and change in percentage of gamma activity before and after LOR was observed (*r* = 0.67, *p* = 0.00012 corrected). Significant correlations are also observed between changes in MSE and changes in delta (*r* = -0.60, *p* = 0.00084 corrected), alpha (*r* = 0.57, *p* = 0.0019 corrected), beta (*r* = 0.74, *p* = 10^-5^ corrected) and gamma power (*r* = 0.64, *p* = 0.00034 corrected). A summary of all of the measured values before and after LOR can be found in **Table 3**.

**Table 2:** Summary of correlations between observed changes before and after loss of response (LOR) and patient demographics.

Parameter 1	Parameter 2	Spearman’s Rho	Significance value, uncorrected
ERR	Age	0.10	0.60
ERR	BMI	0.09	0.66
MSE	Age	-0.28	0.15
MSE	BMI	0.22	0.25

*The Spearman correlation between changes in the ellipse radius ratio (ERR, calculated from the shortest delay 4 ms) and multi-scale entropy (MSE) values before and after LOR (post – pre) with age and body-mass index (BMI) of patients used in the analysis is reported here. No correlations were significant*.

**Table 3:** Summary of correlations between measured changes before and after loss of response (LOR).

Parameter 1	Parameter 2	Spearman’s Rho	Significance value, uncorrected
ERR	MSE	0.54	0.0034^∗^
ERR	Delta	-0.24	0.22
ERR	Theta	0.12	0.53
ERR	Alpha	0.17	0.38
ERR	Beta	0.29	0.13
ERR	Gamma	0.67	0.00012^∗^
MSE	Delta	-0.60	0.00084^∗^
MSE	Theta	0.51	0.0061
MSE	Alpha	0.57	0.0019^∗^
MSE	Beta	0.74	1.09e–05^∗^
MSE	Gamma	0.64	0.00034^∗^

*The Spearman correlation between changes in the ellipse radius ratio (ERR) and multi-scale entropy (MSE) values before and after LOR (post-pre) is reported here. Additionally, Spearman correlation between these values and the changes in percentage of power in individual frequency bands (delta, theta, alpha, beta, and gamma) before and after LOR (post–pre) are reported as well. The ^∗^indicates the correlation between the two parameters was significant at *p* < 0.05 corrected*.

Cohen’s D values revealed medium effect sizes for both complexity measures: sample entropy and ERR (**Table 4**, D_MSE_ = -0.55, D_ERR_ = -0.69). Cohen’s D values were also sizeable for percentage of power differences before and after LOR in alpha and gamma frequency bands (**Table 4**, D_1/_*_f_* = 0.68, D_Alpha_ = 0.61, D_Gamma_ = -0.55). The rest of the frequency bands had small effect sizes (**Table 4**).

**Table 4:** Summary of Cohen’s D for measured values before and after loss of response (LOR).

EEG measure	Cohen’s D
1/*f^α^* fit	0.68
SampEn	-0.55
ERR	-0.69
Delta	-0.20
Theta	0.15
Alpha	0.61
Beta	0.34
Gamma	-0.55

*We calculated Cohen’s D (sign convention: post-pre) to quantify the effect sizes of our EEG measures. We included all measures that showed significant differences before and after LOR, and the standard frequency bands for comparison. ERR value shown here is for the 4 ms delay*.

## Discussion

We found spectral results similar to those reported for propofol anesthesia (Gugino et al., 2001; John et al., 2001; Purdon et al., 2013, 2015a). Specifically, we saw increases in the percentage of alpha activity and decreases in the percentage of gamma activity with LOR. Conversely, we did not observe a significant difference in the percentage of slow (0.1–1 Hz) frequency from before to after LOR, nor did we observe a significant difference in spectral edge frequency before and after LOR. This is likely due to the overall reduced amplitudes of EEG signals in our elderly patients (Purdon et al., 2015b), which reduces the magnitude of the spectral changes. In addition, patients were sedated heavily before they lost consciousness, so EEG changes were subtle. Since we were doing a retrospective data analysis, we were not able to control the initiation of maintenance anesthesia, so some of our patients were on sevoflurane in oxygen with 50–60% nitrous during the post-LOR timepoints. This may have obscured a change in spectral edge frequency as nitrous oxide maintains higher frequencies (Rampil et al., 1998). Sevoflurane, on the other hand, causes similar changes in EEG activity compared to propofol (Akeju et al., 2014; Purdon et al., 2015a). Overall, these nuances between common clinical anesthetics highlight the importance of developing new tools to better distinguish anesthetic states using EEG (Eagleman and Drover, 2018).

One interesting spectral analysis not previously applied to anesthesia EEG is the calculation of the 1/*f* frequency scaling. We chose this measure as it distinguishes different brain states (Bédard et al., 2006) and ages (Voytek et al., 2015). To our knowledge, we have demonstrated the first observation of a change in 1/*f* frequency scaling in EEGs in an anesthesia protocol. 1/*f* frequency scaling was sensitive to before and after LOR.

Previous studies reported differences in CD with anesthetic depth (Widman et al., 2000; Walling and Hicks, 2006), but our study differs in several important aspects. In these earlier studies, patients were anesthetized with sevoflurane to deep levels of anesthesia, but in our study, patients were anesthetized with propofol and fentanyl; further, we limited our analysis to 20 s before and after LOR. Additionally, we used a unique patient population consisting only of geriatric patients with beta-adrenergic blockade. We determined that our CD results were not based on embedding delay or differences between 3 and 5 dimensions.

There are several possibilities as to why we did not find significant differences in CD before and after LOR. For instance, the attractor might be better resolved with higher sampling frequency. Additionally, brain activity might be better represented in an even higher dimensionality embedding. Changes in CD also may not be observable due to age-related changes in complexity (Pierce et al., 2000; Müller and Lindenberger, 2012; Sleimen-Malkoun et al., 2014). The other possibility is that CD might not be sufficiently sensitive to detect the changes that occur during before and after LOR.

We did observe a change in attractor shape similar to what has been previously described (Watt and Hameroff, 1988; Walling and Hicks, 2006; MacIver and Bland, 2014; Eagleman et al., 2018). Additionally, our phase-space analysis based on the geometry of attractors showed significant differences before and after LOR at very short delays (shifting the EEG signal by 1 or 2 points), as previously reported (Eagleman et al., 2018). However, our results demonstrate variability in our population. To determine the source of this variation, better control and measurement of the anesthetics administered in a prospective study is needed. We noticed the magnitude of our results were reduced in the current study compared to our recently published results (Eagleman et al., 2018). This may be due to the current patient population being more sensitive to anesthetics and thus more sedated at our pre-LOR timepoint. We also tested the impact of the embedding delay on our analysis to explore whether changes in EEG signals before and after LOR existed at longer timescales. We found that shorter delays better distinguished before and after LOR and results were no longer significant when the signal was delayed by three points. Visual inspection of the attractor shapes supports this result. Further work is needed to elucidate whether calculations performed in real-time can classify anesthetic depth adequately.

In addition, we tested whether another complexity measure, MSE, could distinguish before- and after-LOR timepoints. We noticed that MSE values appear to converge within a smaller range in the post-LOR period. The distribution suggests that there is one group of patients that show a large decrease in this EEG measure with LOR, while another group does not. The differences between the groups were outside the scope of the current study; however, we plan to examine these differences in the future in a well-controlled prospective study. Additionally, previous reports that showed high correlation of MSE with existing anesthetic depth measures (BIS index and expert anesthesiologist assessment) used EEG data from the entirety of the surgery (induction through recovery), which exposed patients to deeper anesthetic levels than in our study (Liu et al., 2015). Thus, further testing of this analysis on more clinically relevant timepoints is needed.

Since several EEG measures were applied here to an anesthetic dataset including some that have not been explored much previously (ERR), we calculated the Spearman correlation between the changes in EEG measures before and after LOR. We did this for the shortest delay of ERR, MSE, and the percentage of all the individual frequency bands we included. We found a significant correlation with the ERR and MSE values. This is interesting because it suggests that the ERR change, and thus attractor shape change, may be related to changes in the complexity of the signal (revealed by reduction in MSE with anesthesia onset). Additionally, a significant correlation between ERR and MSE with the percentage of gamma change was observed. These relationships are expected as both reductions in gamma activity and reductions in entropy with anesthetic administration have been previously reported (John et al., 2001; Li et al., 2010). The change in MSE is also correlated with changes in the percentages of delta, alpha, and beta power. This again is expected given the ability of MSE to capture all of the spectral changes observed with anesthesia onset (Li et al., 2010).

We tested the effect size of the EEG measures using Cohen’s D. None of these measures had large effect sizes (> 1) indicating the challenge with detecting subtle EEG changes in geriatric patients. ERR and 1/*f* frequency scaling had the largest effect sizes, which were medium in magnitude. This indicates that supplementing complexity measures may improve geriatric patient monitoring. However, further testing of these analyses on more clinically relevant time points and on full EEG traces is needed to test this idea.

As with any study involving EEG, muscle contamination is a potentially important source of artifact. Three of the authors visually inspected all of the EEG clips as well as the processed EEG spectrum and spectrograms to ensure they were free of artifacts. Activity from facial and neck muscles can appear 20 Hz and above, and thus into the frequency ranges we used for analyses (Shackman et al., 2009; Claus et al., 2012; Muthukumaraswamy, 2013); however, as we have reasoned previously (Eagleman et al., 2018), it is important not to throw out higher frequency activity, as it plays an important role in brain-state dynamics (Muthukumaraswamy, 2013), especially in judging anesthetic depth (Sleigh et al., 2001). The measures that we have used have been tested in similar experimental paradigms on intracranial recordings free from EMG contamination (Bédard et al., 2006; MacIver and Bland, 2014; Voytek et al., 2015).

Since these results are only from retrospective analyses our work is limited in several ways. We were not able to control the timing of drug delivery (such as delivery of muscle relaxants); several anesthesiologists with potentially diverse clinical practices were involved, and consciousness measures were restricted to the first loss of response to verbal commands. However, all participating anesthesiologists were instructed to administer anesthesia and a small number of adjuvant agents as per strict protocol guidelines (Drover et al., 2011). Additionally, the protocol of anesthetic and adjuvant agent administration in this retrospective dataset is aligned with current clinical practice. Thus, our results are relevant to current practices of balanced anesthesia administration. Additionally, we tested whether any of our measured results were correlated with patient age or BMI and found no significant correlations. Future prospective work will include several measures to better titrate our analysis to anesthetic action. Collection of blood samples or exhaled vapor can help us correlate results with anesthetic delivery more accurately. Whenever possible, future work should control the delivery of muscle relaxants and the initiation of maintenance anesthetics to separate out the effects of individual anesthetic and adjuvant agents on our measures. Additionally, our results need to be tested on more clinically relevant timepoints, and alongside spectral measures on full EEG traces instead of clips, to better prepare our analyses for clinical application.

Nonetheless, we have observed significant differences before and after LOR using several techniques in a traditionally hard-to-monitor patient group. Future work will discern if these results are useful supplemental tools to better guide physicians in monitoring anesthetic depth in sensitive patient populations. Development of better EEG analysis techniques will hopefully encourage the wide adoption of EEG monitoring and improve the standard of care.

## Conclusion

We found that frontal spectral changes before and after LOR in geriatric patients were limited to the alpha and gamma ranges. Further, we showed that 1/*f* frequency scaling differed before and after LOR. We tested the ability of several measures from nonlinear dynamics, including CD, MSE, and a geometric characterization of time-delayed embeddings, to distinguish LOR timepoints. Among these, MSE and the geometric characterization showed significant differences and had comparable or greater effect sizes to standard frequency measures. In the future, these results may enable the development of better methods of quantifying anesthetic depth in geriatric patients as they are able to significantly discriminate between the subtle EEG changes that occur before and after loss of response.

## Author Contributions

MM, DD, and SE conceived the study. DD acquired the data. SE, DD, and CD organized anesthesia records for analysis. SE and DV analyzed the data. MM, NO, and MC supervised the data analysis. SE and DV wrote the manuscript. All authors contributed to the intellectual content with rounds of review and approved the final version of the manuscript for publication.

## Conflict of Interest Statement

DD is a consultant for Masimo Inc. The original study was conducted with financial support from Hospira, Inc. The remaining authors declare that the research was conducted in the absence of any commercial or financial relationships that could be construed as a potential conflict of interest.

## Footnotes

1 http://chronux.org/
